# Nature, Eco, and Adventure Therapies for Mental Health and Chronic Disease

**DOI:** 10.3389/fpubh.2017.00220

**Published:** 2017-08-21

**Authors:** Ralf Christopher Buckley, Paula Brough

**Affiliations:** ^1^Menzies Health Institute of Queensland, Griffith University, Southport, QLD, Australia

**Keywords:** nature, adventure, outdoor, sport, recreation, psychotherapy, policy making

## Introduction

Many analyses and reviews have concluded that, at least for some individuals in some circumstances, exposure to nature can lead to improvements in multiple mental and physical health parameters and that this applies for both contemplative and adventurous activities (1–15). Over at least the past 4 decades, countries have trialed a wide range of public health programs aimed to increase public participation in outdoor activities, including visits to parks (14–18). At the same time, however, social and technological changes have created opposing pressures: education, work, and lifestyles in developed nations have become increasingly urbanized and indoors (19). Perhaps, as a result, these public health programs have achieved only limited success to date.

This issue is important in public health, since many developed nations are now experiencing increasing social and economic costs from depression, dementia, obesity, and diabetes (10–13, 20–22). These diseases are distinct, but correlated across individuals, and known jointly as chronic disease syndrome (CDS). They are driven partly by genetics (23), but largely by lifestyle (10–13). Older individuals live longer, in poor health, but children are also affected (24). Costs include treatments and healthcare, lost productivity, paid and unpaid carers, and decreased quality of life (QOL) (6, 20–22, 25). In total, these costs may be ~10% GDP for nations with aging populations and high *per capita* healthcare expenditure (6, 20–22, 25).

If we could design health programs or interventions that use outdoor nature-based activities to prevent or treat CDS cheaply and effectively, then that would provide an opportunity to alleviate substantial individual suffering and to overcome a major and growing budgetary problem for national governments (26). A wide range of such programs do exist, under names such as ecotherapies (2), adventure therapies (27), outdoor adventure interventions (27), ecopsychosocial interventions (13), lifestyle therapies (28), and green prescriptions (18, 29, 30), but currently, at rather small scale in global terms. We refer to them here, in aggregate, as nature, eco, and adventure therapies (NEATs). Here, we identify some obstacles to their success and propose research and policy changes for more effective implementation.

We suggest that public and private NEAT programs have been too poorly targeted, and used too small a dose, to prove effective. We propose that this obstacle can be overcome by designing NEATs that are routinely prescribable as a part of clinical healthcare systems. We suggest that while there is ample evidence, as outlined above, that nature exposure and activities can prevent, delay, or alleviate the mental health components of chronic disease, this has been principally at proof-of-concept level. Dose–duration–response relationships, necessary to design practical and prescribable NEATs, remain largely studied (31).

## Research to Underpin Policy

To underpin policy changes, we suggest that research is first required on these dose–duration–response relationships. Short-term effects of NEATs can be measured, for example, through changes in anxiety and stress, attitudes and behavior, efficacy and productivity, and self-reported QOL. We should compare effects of NEATs for (a) different mental health conditions and degrees of severity; (b) different individuals, depending on gender, age, personality, and circumstances; and (c) different therapy types, intensities, frequencies, length of each treatment session, and overall duration of the course of treatment.

Research is also required on techniques to trigger changes in patient lifestyles that continue after an initial prescribed course of NEAT is complete. This requires that patients perceive an improvement in health and happiness during the prescribed course, sufficient to motivate them to continue subsequently. This is a similar model to many physiotherapies and psychotherapies (32, 33). Lifestyle change may require greater dose and duration than improvement during treatment. This research is social rather than clinical. The mechanisms are social levers (34), and the measures of success are behavioral changes, QOL, improved productivity, reduced antisocial behavior, and reduced use of treatment facilities.

Finally, we need research on how the adoption of NEATs, either alone or in conjunction with physical therapies and nutrition, may be influenced by cultural traditions and circumstances. NEATs are limited by cultures and climate (35) as well as by health budgets (20). Countries with easy access to nature, benign climate, and social acceptability of outdoor activities for all demographic groups are ideal for NEATs.

## Policy Options and Implications

Once courses of treatment have been designed and trialed to provide both short- and long-term effectiveness, adoption of NEATs will need changes to the institutional structures of healthcare systems. NEATs meeting criteria for prescription will need to be defined and described in detail. NEAT treatments and providers will need to be certified and licensed. Practitioners will need training in diagnosis, prescription, and evaluation.

Prescribing NEATs through licensed providers involves costs and funding. Currently, many health insurers recommend low-cost patient-funded or publicly funded outdoor activities as a preventive measure, but few support prescribable NEATs as therapeutic measures. Health insurers and government health agencies need to determine what NEATs they will insure or support and what costs they will fund. Not everyone has health insurance, so public funding will be needed for those who do not. This is a good public investment, since NEATs are cheaper than alternatives, and also reduce other public costs such as aged care.

Currently, NEATs are available principally through preventive, public health approaches, targeted only at broad demographic subsectors. Governments advertise their advantages, and urban planners provide opportunities (36), although these are not always socially equitable (37). Curative clinical health approaches, customized to individual symptoms and diagnosed and defined by expert practitioners, are uncommon for NEATs (29, 30). Even once NEATs become prescribable as treatments, they will also remain important preventive components of public health. To make NEATs most effective, we need to target NEATs to individuals most likely to adopt and benefit from them (34).

Introducing prescribable NEATs involves political obstacles and risks. Governments and health insurers will gain from NEATs, but pharmaceutical corporations may lose. Political support for NEATs will be higher in countries where pharmaceuticals are imported, at net public cost. As prescribable NEATs are introduced, legal frameworks will be needed to avoid certified NEAT providers forming oligopolies to exclude competitors and control access to sites.

## Actionable Recommendations

Our principal long-term recommendation is that we should modify healthcare and health insurance systems in developed nations, so as to support routine prescription of certified and insured NEATs for prevention and treatment of the mental health components of CDS. Our short-term and immediately actionable recommendation is that we should conduct research as below to design therapies that are effective, cost-effective, accepted, and adopted.

By using quantitative questionnaire-based approaches, we should test how self-reported QOL, and use of publicly funded mental health treatments, may be correlated with outdoor activities and nature exposure at population scale, when adjusted for geographic location and socioeconomic status; and how self-reported QOL for NEAT participants may differ from overall population averages.

By using qualitative interview-based approaches, we should investigate how individuals engaging in NEATs describe effects on their mental and psychological health. These approaches can be applied for both low-key activities such as visiting parks or beaches and for high-intensity activities involving powerful emotions, e.g., through wildlife encounters or risk recreation (38).

By using experimental interventions, we should test what social levers persuade individuals to increase use of NEATs, for different demographic groups under different social constraints. This includes preschool and school-age children; university students; employees at various types of workplaces; families, including those subject to domestic dysfunction; individuals with disabilities and their carers; and retirees and aged persons and their carers.

By using controlled experimental approaches, we should differentiate the effects of: active (38) verses contemplative (39) types of NEAT; time schedules, such as daily routines, weekends, or intermittent events; places, such as urban greenspace, national parks, and wilderness areas; and guided verses self-paced NEATs.

## Conclusion

We suggest three principal conclusions. First, previous research shows that for at least some individuals and in at least some circumstances, NEATs can improve mental health: a basic therapeutic effect is well demonstrated. Second, attempts to deploy these therapies through public health programs and green prescriptions have not reached their potential, because we lack the evidence required to advance from demonstration of therapeutic effect and to design of effective courses of treatment. Therefore, we identify requirements for research to take this step. Third, once courses of treatment are ready for use as routinely prescribable therapies, changes to healthcare and health insurance systems will be needed to support deployment. However, these changes are relatively minor and are closely analogous to systems already in place for a range of physiotherapies and psychotherapies.

The research program proposed here is substantial, but to use NEATs in preventing and treating mental health components of CDSs, it is both necessary and justified economically. Comparable programs are mandatory for new pharmaceutical treatments. If NEATs can cut the costs of poor mental health by even 1%, that will be a saving of billions of dollars annually in most developed nations. This is an investment well worth making, for both public health research funders and private health insurers.

## Author Contributions

All authors listed have made a substantial, direct, and intellectual contribution to the work and approved it for publication.

## Conflict of Interest Statement

The authors declare that the research was conducted in the absence of any commercial or financial relationships that could be construed as a potential conflict of interest.
